# ﻿*Spiradiclisliboensis* (Rubiaceae), a new species from limestone mountain areas in Guizhou, China

**DOI:** 10.3897/phytokeys.204.84397

**Published:** 2022-08-08

**Authors:** Xiao-Fei Song, Wen-Jian Liu, Ao-Xue Chen, Zheng-Ming Yao, Hong-Bo Lan, Lei Wu

**Affiliations:** 1 College of Forestry, Central South University of Forestry and Technology, Changsha 410004, Hunan, China Central South University of Forestry and Technology Changsha China; 2 Hunan Qingyanghu State-owned Forest Farm, Changsha 410627, Hunan, China Hunan Qingyanghu State-owned Forest Farm Changsha China; 3 Maolan National Nature Reserve, Qiannan 558400, Guizhou, China Maolan National Nature Reserve Qiannan China

**Keywords:** China, limestone, Rubiaceae, *Spiradiclis*, taxonomy

## Abstract

*Spiradiclisliboensis* L. Wu & W. J. Liu, a new species in tribe Ophiorrhizeae of Rubiaceae from limestone mountain areas of Guizhou, south-western China, is described and illustrated. It is similar to *S.guangdongensis* and *S.jingxiensis*, but differs from the latter two by the following traits: stipule triangular, inflorescence sessile or with peduncle up to 0.5 mm long, pedicel 0.8–2.2 mm long, corolla white, salverform, corolla tube 1.6–2.2 cm long, corolla tube of long-styled morph inside with a villous ring and stigmas positioned at the throat of the corolla tube. The conservation status is assessed as “Vulnerable” (VU) according to the IUCN Red List Categories and Criteria.

## ﻿Introduction

*Spiradiclis* Blume, a member of the tribe Ophiorrhizeae (Rubiaceae), is a poorly known and taxonomically complicated genus (Razafimandimbison and Rydin 2019; Tong et al. 2020; Li et al. 2021). There are approximately 59 species worldwide, of which 52 known species are distributed in southern and south-western China (Deb and Rout 1989; Chen and Taylor 2011; Tong et al. 2020; Wen et al. 2021) and these are one of the most representative herbs in limestone areas in the country (Wang 2002; Chen and Taylor 2011; Wen et al. 2019; Wu et al. 2019a; Li et al. 2021).

*Spiradiclis* most closely resembles *Ophiorrhiza* L. and the two genera are in the same tribe Ophiorrhizeae, based on morphological characters (Verdcourt 1958; Darwin 1976; Lo 1999; Chen and Taylor 2011; Wu et al. 2019b) and molecular evidence (Bremer and Manen 2000; Bremer 2009; Rydin et al. 2009; Wikström et al. 2013). Even so, the monophyly of the two genera is questioned (Razafimandimbison and Rydin 2019). However, *Spiradiclis* is morphologically different from *Ophiorrhiza* by its linear-oblong or subglobose capsules with four valves (vs. obcordate and compressed capsules with two valves) when mature. Since the delimitation and relationship of the two genera still need further research, we prefer to accept the traditional concept of *Spiradiclis* here due to its unique capsule form.

During fieldwork in Libo County, Guizhou Province, Chen Yaping (Kunming Institute of Botany, Chinese Academy of Sciences) came across a peculiar population of *Spiradiclis* in flower on a limestone hill and consulted us for identification. This population was initially considered to be *S.guangdongensis* H. S. Lo or *S.jingxiensis* R. J. Wang by its creeping habit, small leaves, 1–2-flowered inflorescences and subglobose capsules. However, after revisiting relevant literature (Lo et al. 1983; Chen and Taylor 2011; Wang et al. 2015; Wen et al. 2015; Wu et al. 2015a, b, 2016, 2019a, b; Pan et al. 2016, 2019; Wang 2016; Liu et al. 2017; Zhang et al. 2018; Wen et al. 2019; Tong et al. 2020; Li et al. 2021), as well as specimens, this population could be distinguished from these two species. Hence, this population is assumed to represent an undescribed new taxon, which is here described.

## ﻿Materials and methods

Materials were deposited at the Herbarium of forest plants in the Central South University of Forestry and Technology (CSFI), Guangxi Institute of Botany, Zhuang Autonomous Region and the Chinese Academy of Sciences (IBK), South China Botanical Garden, Chinese Academy of Sciences (IBSC) (acronyms according to Thiers 2018). Morphological observations and measurements of the new species were based on living material and specimens. Morphological terms follow Harris and Harris (2001).

## ﻿Taxonomic treatment

### ﻿ Spiradiclis liboensis

Taxon classificationPlantaeGentianalesRubiaceae

L. Wu & W. J. Liu sp. nov.

D2558360-D87B-5E16-A082-E1DE7AE94A8E

urn:lsid:ipni.org:names:77303030-1

Figs 1 , 2

#### Type.

China. Guizhou Province: Libo County, Maolan National Nature Reserve, 107°56'E, 22°5'N, alt. 900 m, 9 May 2018 (flower), *L. Wu & F. L. Chen 6410* (holotype: CSFI barcode 069626, isotypes: CSFI, IBK).

**Figure 1.**
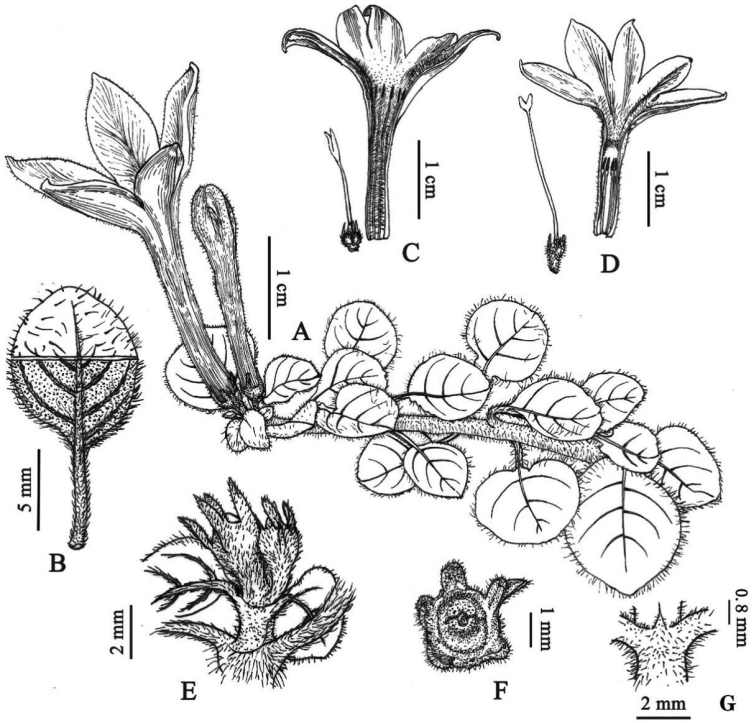
*Spiradiclisliboensis* L. Wu & W. J. Liu. **A** habit **B** leaf blade, adaxial and abaxial views **C** short-styled flower **D** long-styled flower **E** infructescence **F** calyx and disc in face view **G** stipules. Drawn by X.Y. Zeng.

#### Diagnosis.

The new species is similar to *S.guangdongensis* and *S.jingxiensis*, but differs from the former by the triangular (vs. linear), ca. 0.8 (vs. 1–3.5) mm long stipule, 1.6–2.2 (vs. 0.8–1) cm long corolla tube and stigma and anthers positioned at (vs. exserted 5 mm above) the throat of the corolla tube in the long-styled form and short-styled form, respectively, and from the latter by its green (vs. purple) stem, 0.8–2.2 mm (vs. 3–5 mm) long pedicels, white (vs. pink) corolla with tube ca. 1.2 mm wide at the lower part, enlarged at the upper part and ca. 4.8 mm wide at the throat (vs. ca. 2 mm).

**Figure 2.**
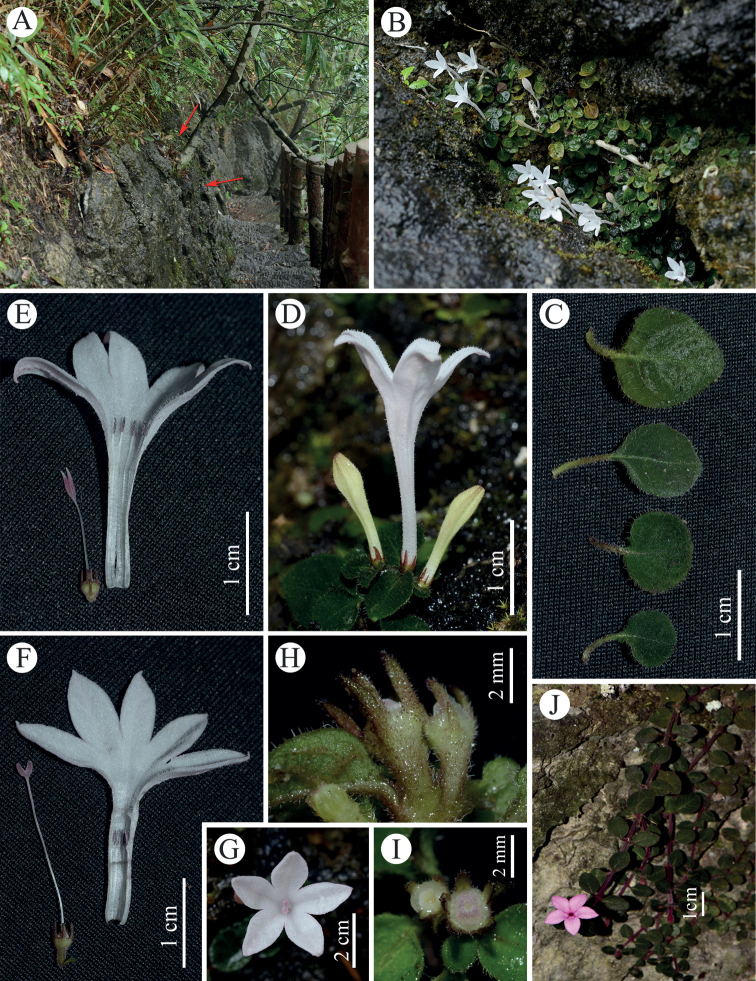
*Spiradiclisliboensis* L. Wu & W. J. Liu. **A** habitat (the arrow shows the place of growth) **B** habit **C** leaves showing variation range **D** inflorescence, side view **E–F** dissected short-styled flower and long-styled flower showing floral parts **G** long-styled flower, frontal view **H** young capsules **I** discs in face view. *S.jingxiensis***J** habit. (Designed by Lei Wu and Xiao-Fei Song).

#### Description.

Perennial herbs, creeping, rooting at most nodes; stems terete, slender, densely pubescent, internodes 1–5 mm long. Petiole 2–9 mm long, sparsely pubescent; leaf blades papery when dry, adaxially green to dark green, abaxially lightly green, ovate to orbicular, 0.3–1.4 × 0.3–1.2 cm, base truncate to rounded, apex subacute to obtuse, adaxially sparsely hispidulous, abaxially pubescent along veins; secondary veins 3–4 on each side; stipules triangular, ca. 0.8 mm long, pubescent, usually deciduous. Inflorescence terminal, cymose, with 1–2 flowers; peduncle 0–0.5 mm long; bracteoles linear or narrowly triangular, 1–2.3 mm long, puberulent. Flowers distylous, 5-merous; pedicels pubescent, 0.8–2.2 mm long. Calyx pubescent; hypanthium obconical, 1.3–1.6 mm long; lobes linear, 1.3–1.8 mm long, outside puberulent. Corolla white, salverform, puberulent outside; tube 1.6–2.2 cm long, ca. 1.2 mm wide at the lower part, enlarged at the upper part, ca. 4.8 mm wide at the throat; lobes subovate, 6–9 × 3–6 mm, puberulent inside; stamens 5; anthers linear-oblong, 1.4–1.6 mm long; stigma bilobed; ovary 2-celled. Long-styled flowers: corolla tube inside with a villous ring below throat and densely pubescent from the throat to the base of the corolla lobes; stamens inserted at the middle of the corolla tube; filaments ca. 0.4 mm long; stigmas positioned at the throat of the corolla tube; lobes elliptic, ca. 1.7 mm long; styles 1.5–1.8 cm long, glabrous. Short-styled flowers: corolla tube pubescent inside; stamens positioned at the throat of the corolla tube; filaments ca. 1.5 mm long; stigmas inserted slightly above the middle of the corolla tube; stigma lobes lanceolate, ca. 3 mm long; styles 7–9 mm long, glabrous. Capsules subglobose, ca. 2.5 mm in diam., pubescent, untwisted valves when mature.

#### Distribution and habitat.

*Spiradiclisliboensis* is currently only known from limestone hills in the Maolan National Nature Reserve, Libo County, Guizhou Province, south-western China. It grows on humid slopes or within crevices under the evergreen broad-leaved forest, at an altitude of 850–950 m. The forest here is dominated by trees of Fagaceae (e.g. *Cyclobalanopsisglauca* (Thunb.) Oerst.), Lauraceae (e.g. *Phoebecalcarea* S. K. Lee et F. N. Wei and *Linderamegaphylla* Hemsl.) and Sapindaceae (e.g. *Handeliodendronbodinieri* (H. Lév.) Rehder).

#### Phenology.

Flowering from May to June, fruiting from June to October.

#### Etymology.

The specific epithet is derived from the type locality, Libo County, southern China. The Chinese name is given as “荔波螺序草” (lì bō luó xù cǎo).

#### Additional specimens examined

**(*paratypes*).** China. Guizhou Province: Libo County, the type locality, 9 May 2019 (fruit), *F. C. Chen & Z. B. Xiong* CFL5029 (IBSC!), 14 October 2019, *F. L. Chen* 19101401 (CSFI!, IBSC!).

#### Provisional conservation status.

After a series of investigations into limestone areas of Guizhou Province, five populations of the new species with approximately 60 individuals at each site have been observed. All the individuals are distributed in Maolan National Nature Reserve and the habitats are mostly in good condition, except for the population adjacent to a tourism road. Hence, according to the IUCN Standards and Petitions Subcommittee (2019) Guidelines, this species is currently evaluated as ‘Vulnerable’ [VU].

#### Discussion.

*Spiradiclisliboensis* is morphologically very similar to *S.guangdongensis* and *S.jingxiensis*. According to the classification of Lo (1999), the three species belong to the subgenus Sinospiradiclis. However, the new species differs from *S.guangdongensis* H. S. Lo mainly by its triangular, shorter stipule, much longer corolla tube and stigma and anthers positioned at the throat of the corolla tube in the long-styled form and short-styled form, respectively, and from *S.jingxiensis* R. J. Wang mainly by its green stem, shorter pedicels, white corolla with tube ca. 1.2 mm wide at the lower part, enlarged at the upper part, ca. 4.8 mm wide at the throat. The detailed morphological comparisons amongst them are listed in Table 1.

**Table 1.** Morphological comparison of *Spiradiclisguangdongensis*, *S.jingxiensis* and *S.liboensis*.

Characters	*Spiradiclisguangdongensis*	*S.jingxiensis*	*S.liboensis*
Petioles length	2–6 mm long	0.5–3 mm long	2–9 mm long
Leaf blades	orbicular; base rounded, apex obtuse, mucronulate or acute	oval to ovate; base broadly cuneate to rounded; apex obtuse to acute	ovate to orbicular, base truncate to rounded
Stipules	linear-subulate, 2–3 mm long	simple or 2-lobed, lobes linear, 1.5–3.0 mm long	triangular, ca. 0.8 mm long
Inflorescence	1–3-flowered, usually one flower	1–2-flowered	1–2-flowered
Peduncle length	Sessile	0–5 mm	sessile or 0.5 mm
Pedicel length	3–7.5 mm	3–5 mm	0.8–2.2 mm
Corolla	white, slender funnelform	pink, salverform	white, salverform
Corolla tubes	0.8–1 cm long, 0.8–2 mm wide, with almost the same width from base to top	1.3–1.9 cm long, ca. 2 mm wide, with almost the same width from base to top	1.6–2.2 cm long, ca. 1.2 mm wide at lower part, enlarged at upper part, ca. 4.8 mm wide at the throat
Corolla lobes	lobes 4–7 × ca. 3 mm	oval, ca. 5.5–6.5 × 2.5–3.0 mm	subovate, 6–9 × 3–6 mm
Position of the stigma and anthers	exserted 5 mm above the throat of corolla tube in long-styled form and short-styled form, respectively	positioned at the throat of corolla tube in both forms	positioned at the throat of corolla tube in both forms
Styles in short-styled form	6–10 mm long	ca. 4.5 mm long	7–9 mm long
Flowering	March to April	May to June	May to June

Currently, there are nine other species of *Spiradiclis* with creeping or decumbent habits and small leaf blades shorter than 3 cm, viz., *S.danxiashanensis* R. J. Wang, *S.glandulosa* L. Wu & Q. R. Liu, *S.hainanensis*, *S.lui* Liu Yan & L. Wu, *S.karstana* L. Wu, X. Li & Q. R. Liu, *S.pauciflora* L. Wu & Q.R. Liu, *S.tubiflora* L. Wu, B. M. Wang & B. Pan, *S.pengshuiensis* B. Pan & R. J. Wang and *S.umbelliformis* H. S. Lo (Chen and Taylor 2011; Wang et al. 2015; Wu et al. 2015a, b, 2019b; Zhang et al. 2018). To better differentiate these species, a key is provided.

### ﻿Key to the species of *Spiradiclis* with stems creeping or decumbent and leaf blades shorter than 3 cm

**Table:** 

1	Calyx lobes ca. 4–6 mm long, oblong-lanceolate	***S.glandulosa***
–	Calyx lobes shorter than 3.5 mm, linear, triangular or subulate	**2**
2	Corollas tubular-funnelform with tubes distinctly enlarged	***S.tubiflora***
–	Corolla funnelform or salverform, usually with a slender tube	**3**
3	Leaf blades usually shorter than 1.5 cm; inflorescences 1–3-flowered	**4**
–	Leaf blades usually longer than 1.5 cm (except *S.pauciflora*); inflorescence many-flowered (more than 3 flowers)	**7**
4	Stigmas and anthers distinctly excluded in long-styled and short-styled forms	**5**
–	Stigmas and anthers included in long-styled and short-styled forms	**6**
5	Inflorescence 2–3-flowered; corolla tube 1.1–1.5 cm long	***S.danxiashanensis***
–	Inflorescence usually 1-flowered; corolla tube 0.8–1 cm long	***S.guangdongensis***
6	Peduncles longer than 5 mm; long-styled corolla inside without a ring of long hairs	**7**
–	Inflorescences sessile or peduncles shorter than 5 mm; long-styled corolla inside with a ring of long hair	**8**
7	Leaf blade usually cordiform-orbicular, base cordulate to truncate	***S.hainanensis***
–	Leaf blade ovate, base cuneate or broadly cuneate, decurrent	**9**
8	Stem green; pedicels 0.8–2.2 mm long; corolla white; corolla tube ca. 1.2 mm wide at lower part, enlarged at upper part, and ca. 4.8 mm wide at the throat; styles 7–9 mm long in short-styled form	***S.liboensis***
–	Stem purple; pedicels 3–5 mm long; corolla pink; corolla tube ca. 2 mm wide, cylindrical, with almost the same width from base to top; styles 4.5 mm long in short-styled form	***S.jingxiensis***
9	Stipules shorter than 2 mm, usually deciduous	**10**
–	Stipules 5–10 mm long, persistent	**12**
10	Corollas funnelform, tubes 7–9 mm long	***S.pauciflora***
–	Corollas salverform, tubes longer than 9 mm	**11**
11	Secondary veins 5–12 pairs; corolla tube 15–25 mm long with a ring of long hairs inside in long-styled form	***S.karstana***
–	Secondary veins 4–6 pairs; corolla tube 9–15 cm long without a ring of long hairs inside in long-styled form	***S.pengshuiensis***
12	Stems densely multicellular villosulous; bracts shorter than 1 mm	***S.umbelliformis***
–	Stems glabrous; bracts 5.5–8.5 mm long	***S.lui***

## Supplementary Material

XML Treatment for Spiradiclis liboensis

## References

[B1] Bremer B (2009) A review of molecular phylogenetic studies of Rubiaceae. Annals of the Missouri Botanical Garden 96(1): 4–26. 10.3417/2006197

[B2] Bremer B Manen J (2000) Phylogeny and classification of the subfamily Rubioideae (Rubiaceae). Plant Systematics and Evolution 225(1–4): 43–72. 10.1007/BF00985458

[B3] Chen T Taylor CM (2011) *Spiradiclis*. In: Wu ZY Raven PH Hong DY (Eds) Flora of China.Vol 19. Science Press, Beijing & Missouri Botanical Garden Press, St. Louis, 330–339.

[B4] Darwin SP (1976) The Pacific species of *Ophiorrhiza* L. (Rubiaceae). Lyonia 1: 48–101.

[B5] Deb DB Rout RC (1989) Two new species of the genus *Spiradiclis* (Rubiaceae) from India. Candollea 44: 225–229.

[B6] Harris JG Harris MW (2001) . Plant Identification Terminology: An Illustrated Glossary. Spring Lake Publishing, 1–188.

[B7] IUCN Standards and Petitions Subcommittee (2019) Guidelines for using the IUCN Red List Categories and Criteria. Version 14. Prepared by the Standards and Petitions Committee. http://www.iucnredlist.org/documents/RedListGuidelines.pdf [accessed 4 October 2020]

[B8] Li JL Yuan Q Liu Y Song XF Pan B Qu CH Wu L (2021) Two new species of *Spiradiclis* (Rubiaceae) from limestone areas in southwestern China. Nordic Journal of Botany 39: e02979. 10.1111/njb.02979

[B9] Liu J Pan B Li SW Xu WB (2017) *Spiradiclisquanzhouensis* (Rubiaceae): A new species from limestone area in Guangxi, China. Nordic Journal of Botany 36(3): e01595. 10.1111/njb.01595

[B10] Lo HS (1999) Spiradiclis Blume. In: Lo HS (Ed.) Flora Reipublicae Popularis Sinicae.Vol. 71 (1). Science Press, Beijing, 86–110.

[B11] Lo HS Sha WL Chen XX (1983) A revision of the genus *Spiradiclis* Bl. Acta Botanica Austro Sinica 1: 27–36.

[B12] Pan B Ma HS Wang RJ (2016) *Spiradiclispengshuiensis* (Ophiorrhizeae, Rubioideae), a new species from Chongqing, China. PhytoKeys 63: 41–45. 10.3897/phytokeys.63.8016 PMC4956927 27489477

[B13] Pan B Tu RH Hareesh VS Wu L (2019) *Spiradicliscavicola* (Rubiaceae), a new species from limestone caves in south-western China. Annales Botanici Fennici 56(1–3): 1–4. 10.5735/085.056.0101

[B14] Razafimandimbison SG Rydin C (2019) Molecular-based assessments of tribal and generic limits and relationships in Rubiaceae (Gentianales): Polyphyly of Pomazoteae and paraphyly of Ophiorrhizeae and *Ophiorrhiza*. Taxon 68(1): 72–79. 10.1002/tax.12023

[B15] Rydin C Kainulainen K Razafimandimbison SG Smedmark JEE Bremer B (2009) Deep divergences in the coffee family and the systematic position of Acranthera. Plant Systematics and Evolution 278(1–2): 101–123. 10.1007/s00606-008-0138-4

[B16] Thiers B (2018) Index Herbariorum: A global directory of public herbaria and associated staff. New York Botanical Garden’s Virtual Herbarium. http://sweetgum.nybg.org/ih/ [accessed 26 June 2021]

[B17] Tong YH Xia NH Wu L Vu TC (2020) Critical notes on *spiradiclis purpureocaerulea* H.S. Lo (Rubiaceae) from vietnam. Adansonia 42(19): 291–296. 10.5252/adansonia2020v42a19

[B18] Wang RJ (2002) Two new species of *Spiradiclis* (Rubiaceae) from China. Novon 12(3): 420–423. 10.2307/3393092

[B19] Wang RJ (2016) *Spiradiclisjingxiensis* sp. nov. (Rubiaceae) from Guangxi, China. Nordic Journal of Botany 34(5): 550–552. 10.1111/njb.01134

[B20] Wang RJ Wen HZ Deng SJ Zhou LX (2015) *Spiradiclisdanxiashanensis* (Rubiaceae), a new species from south China. Phytotaxa 206(1): 30–36. 10.11646/phytotaxa.206.1.5

[B21] Wen HZ Wang RJ Deng SJ (2015) *Spiradiclislonganensis*, a new species of Rubiaceae from China. PhytoKeys 55: 113–117. 10.3897/phytokeys.55.4975 PMC4547028 26312046

[B22] Wen ZJ Yang JC Xu YF Wu L (2019) *Spiradiclisdensa* sp. nov. (Rubiaceae) from limestone areas in Guangxi, China. Nordic Journal of Botany 37(6): e02190. 10.1111/njb.02190

[B23] Wen ZJ Huang YF Hu YH Nguyen KS Wu L (2021) *Spiradiclisdetianensis* (Rubiaceae, Ophiorrhizeae), a new species from south-western Guangxi, China. PhytoKeys 184: 103–110. 10.3897/phytokeys.184.69886 34785974 PMC8589774

[B24] Wikström N Neupane S Kårehed J Motley TJ Bremer B (2013) Phylogeny of *Hedyotis* L. (Rubiaceae: Spermacoceae): redefining a complex Asian-Pacific assemblage. Taxon 62(2): 357–374. 10.12705/622.2

[B25] Wu L Wang JL Mo SS Liu QR (2015a) *Spiradiclisglandulosa* sp. nov. (Rubiaceae) from limestone areas in southern China. Nordic Journal of Botany 33(1): 79–82. 10.1111/njb.00577

[B26] Wu L Wang JL Liu QR (2015b) *Spiradiclispauciflora* (Rubiaceae), a new species from limestone areas in Guangxi, China. Annales Botanici Fennici 52(3–4): 257–261. 10.5735/085.052.0318

[B27] Wu L Tong Y Pan B Liu QR (2016) *Spiradiclisglabra* sp. nov. (Rubiaceae) from limestone areas in Guangdong, China. Nordic Journal of Botany 34(6): 718–721. 10.1111/njb.01156

[B28] Wu L Li X Liu WJ Liu QR (2019a) *Spiradicliskarstana* (Rubiaceae), a new species from Yunnan, China. PhytoKeys 117: 1–8. 10.3897/phytokeys.117.28281 PMC6370749 30766422

[B29] Wu L Wang BM Pan B Yu XL (2019b) *Spiradiclistubiflora* (Rubiaceae), a new cave-dwelling species from southern China. PhytoKeys 130: 217–224. 10.3897/phytokeys.130.34625 31534408 PMC6728394

[B30] Verdcourt B (1958) Remarks on the calassification of the Rubiaceae. Bulletin van den Rijksplantentuin, Brussel 28: 209–281.

[B31] Zhang F Liu Y Wen ZJ Wu L (2018) *Spiradiclislui*, a new species of Rubiaceae from Guangxi, China. Nordic Journal of Botany 36(6): e01786. 10.1111/njb.01786

